# Inclusions of Pesticides by β-Cyclodextrin in Solution and Solid State: Chlorpropham, Monuron, and Propanil

**DOI:** 10.3390/molecules28031331

**Published:** 2023-01-30

**Authors:** Martina Dragone, Getasew Shitaye, Gianluca D’Abrosca, Luigi Russo, Roberto Fattorusso, Carla Isernia, Gaetano Malgieri, Rosa Iacovino

**Affiliations:** 1 Department of Environmental, Biological and Pharmaceutical Sciences and Technologies, University of Campania “Luigi Vanvitelli”, Via Antonio Vivaldi 43, 81100 Caserta, Italy; 2 Department of Biomedical Sciences, School of Medical Sciences, Bahir Dar University, Bahir Dar 6000, Ethiopia

**Keywords:** inclusion complex, β-Cyclodextrin, pesticides, UV-Vis spectroscopy, molecular docking

## Abstract

Persistence and degradation are important factors in determining the safe use of such synthetic products, and numerous studies have been addressed to develop pesticide remediation methods aimed at ameliorating these features. In this frame, the use of different cyclodextrins (CDs) molecules has attracted considerable attention due to their well-known non-toxic nature, limited environmental impact, and capability to reduce the environmental and health risks of pesticides. CDs appear to be a valuable tool for the elimination of pesticides from polluted areas as well as for better pesticide formulations that positively influence their hydrolysis or degradation. The present work investigates the interaction between β-cyclodextrins and three commonly used pesticides (i.e., chlorpropham, monuron, and propanil) both in solution and in the solid state by means of UV-Vis, FT-IR, and X-ray powder diffractometry. We show that such interactions result in all three cases in the formation of inclusion complexes with a 1:1 stoichiometry and binding constants (K_b_) of 369.9 M^−1^ for chlorpropham, 292.3 M^−1^ for monuron, and 298.3 M^−1^ for propanil. We also report the energy-minimized structures in silico for each complex. Our data expand and complement the available literature data in indicating CDs as a low-cost and very effective tool capable of modulating the properties that determine the environmental fate of pesticides.

## 1. Introduction

Pesticides are a wide group of organic compounds that bear different physicochemical properties and, as such, are a pillar in pest management. Their large and increasing use, with millions of tons of synthetic pesticides used worldwide every day in intensive agricultural productions, along with their persistence and pervasiveness, has led to the fact that almost every ecosystem has been negatively impacted by them [1,2]. In many cases, the chemicals are not completely selective to the pest, raising the probability of causing adverse effects on human health and other non-target organisms. Most pesticides also have the disastrous property of being strongly adsorbed onto soil organic matter that further slows their degradation in the environment. Polluting agents, including pesticides, on top of their resistance to degradation, often have increased toxicity due to their ability to kill detoxifying microorganisms. As a consequence, the risks for human and ecosystem health are becoming more and more pressing [3,4].

Humans can be exposed to pesticides in multiple ways, including dermal contact, inhalation, and ingestion. The way and time of exposure, in turn, determine the degree of toxicity for each pesticide, which can range from mild symptoms such as minor allergies or slight skin irritation to more severe diseases [5,6]. Many studies have collectively shown acute and chronic health effects of pesticides: damage to the human genome, chronic-degenerative disorders, immune dysregulation, reproductive toxicology, and developmental problems [7,8]. In vitro [9], animal [10], and human [11] investigations evidenced epigenetic modification by pesticides. Indeed, direct exposure to pesticides is the foremost reason for multiple forms of cancer globally [12], while their relationships with Parkinson’s disease, autism spectrum disorder, and other neurodevelopmental disorders are intensively studied. Although the effects of exposure to pesticides differ from person to person, for multiple reasons, pregnant women and children are more susceptible [13,14].

**Figure 1:**
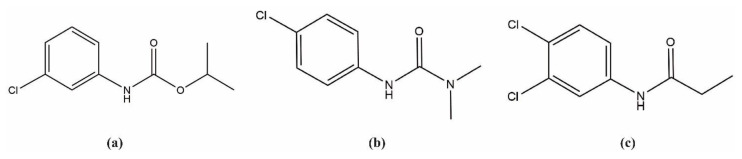
Chemical structure of (**a**) Chlorpropham; (**b**) Monuron; (**c**) Propanil.

The three common pesticides studied in this article, namely propanil (3,4-dichloroproprioanilide), monuron (*N*’-(*p*-chlorophenyl)-*N*,*N*-dimethylurea), and chlorpropham (isopropyl-3-chlorophenyl carbamate), are grouped under aniline, phenylurea and carbamate herbicides, respectively (Figure 1). These compounds are associated with negative effects on environmental and human health. There is evidence of a strong relationship between propanil poisoning and methemoglobinemia and hemolysis [15,16]. Concerns have also been raised about monuron toxicity and carcinogenicity. It has been shown to induce chronic renal toxicity [17] and transcriptomic alteration [18] in rats, while its potential endocrine-disrupting effects [19] have been reported through in vitro studies. Chlorpropham, instead, has been linked to potentially adverse effects on human health in the long term, although the likelihood of exposure to it seems low. Experimental studies on mice evidenced the reproductive and neurobehavioral effects [20] and mitochondrial dysfunction of this pesticide [21], which also has DNA binding properties [22].

For these reasons, in the last couple of decades, a significant number of methods have been applied for remediation purposes. Along with a plethora of eco-friendly remediation techniques that are being developed and utilized, cyclodextrins (CDs) gained significant attention due to their ability to modulate pesticide properties [23,24]. Numerous studies revealed the effectiveness of CDs used as complexation and extraction agents for pesticides [25,26]. One main disadvantage of synthetic pesticides is their low water solubility. Though the solubility depends on the concentration, CDs and their derivatives are considered agents for improving the water solubility of some pesticides [27]. Additionally, CD application has the advantage of overcoming inherent drawbacks of organic solvent and surfactant use. Noteworthy, from a toxicological point of view, CDs are not associated with significant side effects on human health. In general, their structural, chemical, and biological stability, along with their ecological viability, make these complexing agents excellent candidates as extraction tools [28].

They are ring-shaped oligosaccharides naturally obtained through the degradation of starch by glucanotransferase enzyme [29] in the form of α-CD, β-CD, and γ-CD with six, seven, and eight glucopyranose units, respectively [30]. They show a low polarity cavity which, in an aqueous solution, is occupied by energetically disfavored water molecules that can be easily substituted by guest molecules resulting in modifications to the physicochemical property of the guest compound [31,32,33,34]. This peculiarity makes CDs suitable for applications in environmental protection by trapping a wide range of pollutant agents. More specifically, the diameter of the β-CDs cavity offers the most suitable size for different compounds [35,36]. Although β-CDs show a lower water solubility compared to other natural and derivative cyclodextrins [37,38,39], they are considered a perfect tool in environmental remediation processes for their low cost [40]. In light of these considerations, it is worthwhile to characterize β-CDs inclusion complex with different pesticides further. To this aim, we herein report the stoichiometry and binding constants of the three mentioned pesticides with β-CDs in solution using UV-Vis spectroscopy along with molecular docking studies. We also report the characterization of the products obtained from the complexation reactions between pesticides and beta cyclodextrin in solid state, using FT-IR, and X-ray powder diffractometry.

## 2. Results

### 2.1. Characterization of Inclusion Complexes in Solution

Enclosure by the hydrophobic CD cavity is accompanied by substantial changes in the guest’s physicochemical properties. This behavior is exploited in the study of the formation of inclusion complexes as it results in changes in the absorbance in the UV-Vis spectrum, which are directly related to the complex concentration [41].

For each pesticide, we determined the stoichiometry of their complexes with β-CD by means of the Job method: we measured the UV-Vis absorbance of a series of samples in which we continuously varied the molar fraction of β-CD and pesticide [42]. According to such a method, also known as the continuous variation method [43], the complexation stoichiometry is represented by the molar ratio R corresponding to the maximum concentration of the complex.

**Figure 2:**
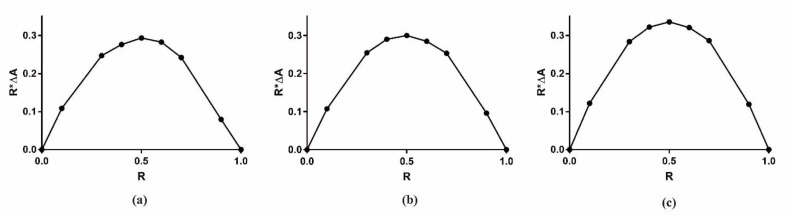
Job plot for the complex: (**a**) Chlorpropham: β-CD (λ = 237.0 nm); (**b**) Monuron: β-CD (λ = 245.0 nm); (**c**) Propanil: β-CD (λ = 250.0 nm). ΔA is the absorbance difference of the pesticide in the absence and in the presence of β-CD.

The maximum of the curve was obtained for all three complexes with R = 0.5, indicating that the three pesticides are included by the β-CD in a 1:1 stoichiometry (Figure 2).

The determination of the host–guest stoichiometry is correlated with the evaluation of the binding constants (K_b_s) of the studied interaction. The Job’s method reports only on stoichiometry, while to estimate K_b_s, it is necessary to monitor changes of an experimental feature directly correlated with the concentration of the guest upon gradual host addition.

**Figure 3:**
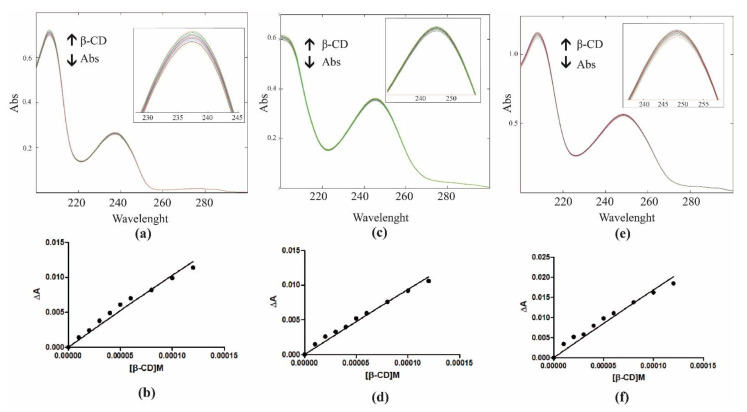
UV-Vis characterization: dependence of chlorpropham (**a**), monuron (**c**), and propanil (**e**) absorbance (Abs) with increasing concentration of β-CD at pH 7.1. The binding constants of β-CD with chlorpropham (**b**), monuron (**d**), and propanil (**f**) were determined by fitting data according to the equation reported in Materials and Methods.

Thus, UV-Vis spectroscopy was also used to evaluate the K_b_s to evaluate the analytical differences between the free and complexed pesticides [44]. In Figure 3, the results of the dependence of pesticides absorbance upon β-CD addition are shown; the maximum absorption wavelengths were found at 237.0 nm for chlorpropham, 245.0 nm for monuron, and 250.0 nm for propanil.

In order to have an accurate determination of the K_b_s (reported in Table 1, data were fitted using a non-linear regression [45,46,47].

The magnitude of the binding constant is an important value when evaluating the inclusion of a specific pesticide in CDs. A large value leads to a complex that does not dissociate, and this, in turn, can result in the inhibition of the pesticide action. On the other hand, the same value could give advantages by changing the half-life of the pesticide, thus extending its action. It is challenging to know what an optimum value for the association constant is as it largely depends on the nature of the final application desired for the obtained complex. The values reported in Table 1 lie well below 10,000 M^−1^, the upper limit in pharmaceutical applications of CD-drug complexes [48], allowing us to speculate a ready release of the included guests from the CD-pesticides complexes here reported in agrochemical applications.

### 2.2. Characterization of Inclusion Complexes in Solid State FT-IR Spectroscopy

**Figure 4:**
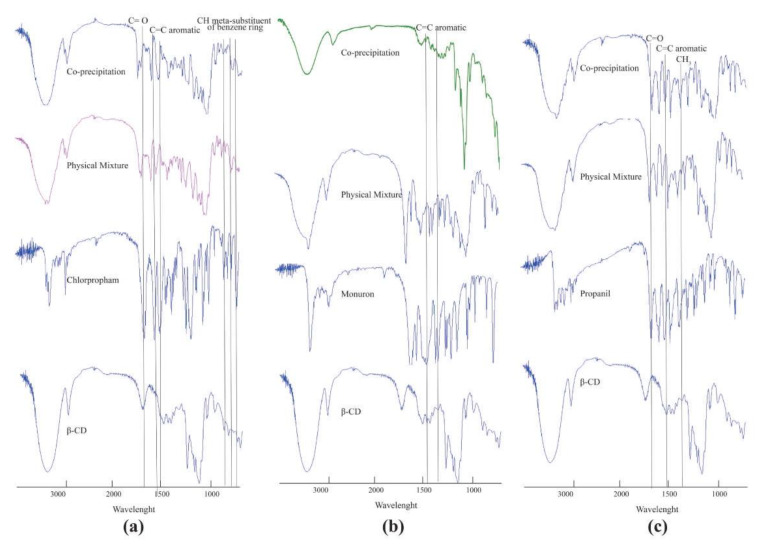
FT-IR spectra recorded for the co-precipitation complexes of Chlorpropham (**a**), Monuron (**b**), and Propanil (**c**) with β-CD are compared, the same spectra recorded for the physical mixture of each pesticide with β-CD and each pesticide or the β-CD alone.

FT-IR Spectroscopy was used to investigate the formation of the inclusion complexes of β-CD with each pesticide. The complexes were produced by means of the co-precipitation method. In order to confirm the formation of the inclusion complexes for each pesticide, the spectrum of the co-precipitation product was compared to that of a physical mixture, that of the β-CD, and the pesticide alone. In all the cases, while the spectrum of the physical mixture appears to be only the simple sum of the individual pattern of signals of each individual compound, the spectra of the co-precipitation products showed changes that indicate the formation of the inclusion complexes (Figure 4 and Table 2).

#### 2.2.1. Chlorpropham: β-CD

In this case (Figure 4a and Table 2), in the co-precipitation product, the characteristic carbonyl stretching band of the pesticide appeared to shift along with the reduced intensity of the same band. This shift suggests a modification of the electronic environment of the C=O group, which is in agreement with the increase generally observed in the frequency of a specific peak of complexation [49]. Changes in the characteristic band of pure pesticide confirm the existence of the complex as a new compound with different spectroscopic bands. The lack of C=O shift of chlorpropham in the spectrum of the physical mixture confirmed that, in this case, no host–guest interaction was observed. Bands included between 1450.00 and 1550.00 nm, characteristic of the aromatic C=C bonds, are clearly perturbed by the presence of the CD moiety. In particular, it is possible to appreciate a clear shift for the band belonging to the pesticide-free form from 1542.40 nm to 1523.42 nm when in complex with the CD, along with a remarkable intensity reduction in the band at 1482.0 nm. The bands for the meta substituents of the aromatic ring can be found in the edge region of the spectrum; also, the pesticide band at 773.68 nm, which represents the C–Cl bond, appears shifted at 761.11 nm and shows a remarkable intensity reduction, indicating its involvement in interactions with the CD [50,51].

#### 2.2.2. Monuron: β-CD

In this case (Figure 4b and Table 2), the main variations in the FT-IR spectra that can be evidenced upon complexation concern the bands assigned to the C=C bonds of the pesticide aromatic ring. These bands show changes in terms of wavelengths and intensity, clearly indicating a change in the chemical environment that surrounds the aromatic ring of monuron.

#### 2.2.3. Propanil: β-CD

In Figure 4c, is shown the intensive band of the carbonyl for propanil that shifted from 1670.29 nm to 1667.98 nm for the product of co-precipitation. The result suggests a modification of the electronic environment of the C=O group, which means that inclusion complexes are formed in the solid state. No shift of this peak was found in the physical mixture. Other perturbations can be evidenced for the aromatic C=C and the ring substituents (CH bending of methyl group) as in Table 2.

### 2.3. Powder X-ray Diffraction

**Figure 5:**
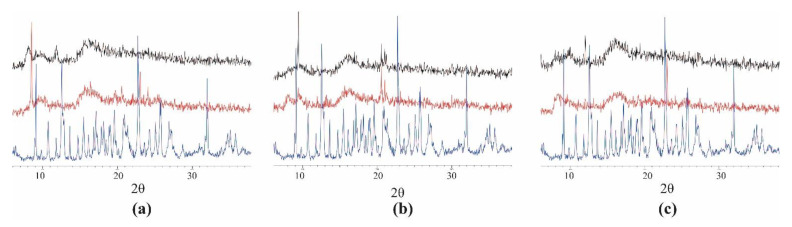
The powder XRD patterns of (**a**) Chlorpropham, (**b**) Monuron, (**c**) Propanil. Black is the co-precipitation complex, red is the pesticide, and blue is the β-CD.

Powder X-ray diffraction (XRD) diffraction studies are useful to allow the identification of the inclusion complex based on the fact that the crystallinity of the compounds changes upon host–guest interaction. XRD patterns of powder diffraction is a useful technique to verify the formation of the inclusion complexes. The crystallinity of the compounds changes upon host–guest interaction, and significantly different X-ray diffraction patterns are to be expected. The powder diffractogram for the β-CD presents diffraction angles of 2θ at 9.08°; 10.71°; 12.64°; 14.72°; 17.11°; 21.09°; 22.80°; 24.23°; 31.96°; which are indicative of its crystalline character. Sharp peaks over the diffraction angles indicate the crystal nature of β-CD and chlorpropham (Figure 5a). On the other hand, a total amorphization was observed for the co-precipitation product due to the disruption of the cyclodextrin lattice by chlorpropham molecules; indeed, the diffraction pattern of the inclusion complex reveals two broad peaks and many undefined, diffused peaks of low intensities, reflecting its amorphous nature.

In the case of monuron (Figure 5b), the formation of the inclusion complex is confirmed by the variations observed between the diffractograms of the pesticide alone and the complexed one. The pesticide pattern contains a series of sharp signals descriptive of its crystallinity at 2θ values of 20.56° and 21.04°. In the complex diffraction pattern, the intensity of such peaks is clearly lower, while the intensity of the signal at 9.45° is clearly higher, indicating a rearrangement of the molecule due to the interaction with the β-CD whose peaks are not visible in the spectrum of the complex.

In the X-ray diffractogram of the propanil powder (Figure 5c), sharp peaks at a diffraction angle of 2θ of 8.03° and 22.99° are present and suggest that the compound is present as a crystalline material. A total pesticide amorphization was instead induced by co-precipitation, where X-ray diffraction patterns of the complex were characterized only by large diffraction peaks in which it is no longer possible to distinguish the characteristic peaks of the pesticide. These results confirm that propanil is no longer present as crystalline material and that its β-CD solid complex exists in an amorphous state.

### 2.4. Molecular Docking

**Figure 6:**
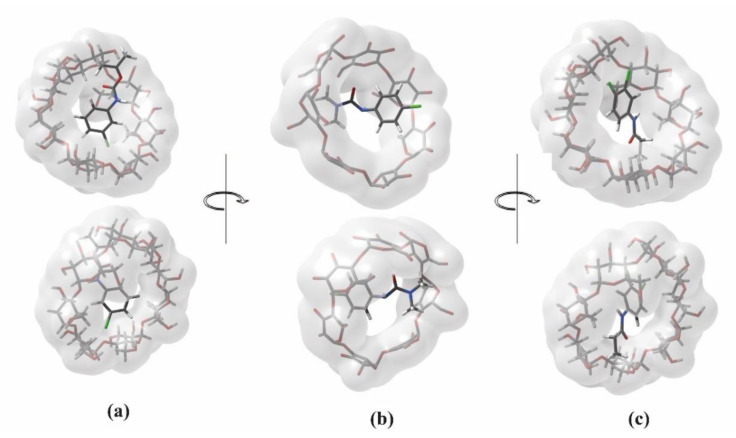
Molecular docking models of (**a**) Chlorpropham, (**b**) Monuron, and (**c**) Propanil in complex with the β-CD.

Molecular Docking represents a powerful tool to characterize the binding mode of an inclusion complex formed by two or more molecules with known structures. For this reason, we performed a series of molecular docking studies to better describe the structural features of the inclusion complexes of the β-CD with the studied pesticides. The adopted molecular docking protocol can be described as follow: the 3D structure of cyclodextrin was downloaded by the protein data bank (PDB), while the compounds were found in the ChEBI (Chemical Entities of Biological Interest, https://www.ebi.ac.uk/chebi, accessed on 17 November 2022) database; all the structures were protonated, energetically minimized and then used as input for the docking simulations, using the parameters described in the Materials and Methods section. The selected models for the β-cyclodextrin in complex with propanil, chlorpropham, and monuron are reported in Figure 6. In the case of propanil’s complex, the small molecule enters the cyclodextrin cavity and extrudes from the tighter rim with the methyl terminal group. The benzene ring is exposed from the wider rim, and the complex is stabilized by the formation of four hydrogen bonds: the propanil amide group interacts with an OH of the CD and with the oxygen of a glucopyranose ring, its carbonyl with an alcoholic group of the CD, and its aromatic ring with the ether oxygen of the CD sugar. The CD includes the monuron giving a complex similar to the one previously described here, with the tail exposing from the tighter rim and the aromatic from the wider ring. However, in this case, the binding appears to be stabilized exclusively by hydrophobic interactions, as no major hydrogen bonds are formed. On the contrary, the chlorpropham exposes the tail from the wider rim, with the aromatic ring placed in the middle of the cyclodextrin cavity. The complex is stabilized by six H-bonds involving the oxygens of the molecule and the alcoholic groups of the CD.

## 3. Materials and Methods

Reagents and solvents of analytical grade, as well as double-distilled and MilliQ water, were utilized throughout the experiments. A 1.0 mM phosphate buffer was prepared at a pH of 7.12 in deionized water. Pesticides, supplied by Dr. Ehrenstofer, were dissolved in this phosphate buffer to produce concentrations of approximately 0.05 mM. The pH values of the buffers were kept constant and continuously checked by using a calibrated CRISON pH-meter Basic 20 (Crison Instruments, Barcelona, Spain).

### 3.1. Determination of Binding Constants by UV-Vis Spectroscopy

Binding constants for the complexes under study were estimated using the molar ratio titration method [52]. A buffered solution of pesticide 0.05 mM was slowly titrated with a β-CD solution at a varied concentration (from 0 to 0.15 mM). K_b_s can be accurately evaluated by direct spectroscopic methods that rely on analytical differences between the free compound and its complexed form. Thus, changes in the absorption intensity of the pesticide were monitored as a function of cyclodextrin concentrations; the maximum absorption wavelength of chlorpropham was found at 237.0 nm, monuron was found at 245.0 nm, and propanil was found at 249.0 nm. To conveniently calculate the *K_b_*, we rearranged the Benesi–Hildebrand Equation into [46]:

(1) $$\Delta A=\frac{\left[\beta -CD\right]\cdot K_{b}\cdot \varepsilon \cdot \left[pesticide\right]}{1+K_{b}\cdot \left[\beta -CD\right]}$$

where Δ*A* is the absorbance difference of the peptide in the absence and the presence of β-CD, [*β − CD*] is the concentration of β-CD, *K_b_* is the binding constant, *ε* is the molar extinction coefficient and [*pesticide*] is the concentration of the pesticide. The binding constants were obtained from the titration curve data Δ*A* as a function of [*β − CD*] fitted by non-linear regression (GraphPad Prism 7 software).

### 3.2. Stoichiometry Determination Using Job Plot Method

Complex stoichiometry was determined using the Job method or continuous variation method [42]. Briefly, unbuffered solutions of pesticide and β-CD at 0.01 mM each were mixed at different molar ratios $R=\frac{\left[pesticide\right]}{\left[pesticide\right]+\left[\beta -CD\right]}$. The volume was kept constant. The stoichiometric ratio of each complex was found as the maximum R of the curve obtained by plotting (ΔA × R) against R; ΔA is the absorbance difference of the pesticide in the absence and in the presence of β-CD. All measurements were conducted at room temperature and collected in the 200–400 nm wavelength range with a Shimadzu UV-1800 spectrometer (Shimadzu, Tokyo, Japan) in a 1 cm quartz cuvette.

### 3.3. Preparation of Inclusion Complexes Pesticides: β-CD in Solid State

The co-precipitation method was used to prepare the complexes in the solid state. A saturated solution of β-CD was obtained using the minimum amount of distilled water. Each pesticide was added to the solution under stirring at 1:2 molar ratios and left under agitation at 60 °C for the necessary time to obtain a homogeneous solution. A white precipitate, obtained by cooling the solution at room temperature overnight, was filtered and washed with chloroform and methanol. After the washing procedure, samples were dried in an oven at 100 °C. A physical mixture was obtained by blending pesticides and β-CD powders in an agate mortar to gain a homogeneous mixture.

For the preparation of chlorpropham β-CD, 0.0376 g of chlorpropham was added under agitation to a solution of β-CD (0.1 g) in water (25 mL) previously warmed to 60 °C. Stirring was maintained for three hours at 60 °C. For the preparation of monuron β-CD, 0.0349 g of monuron was added while stirring to a solution of β-CD (0.1 g) in water (25 mL) previously warmed to 60 °C. Stirring was maintained for two hours and thirty minutes. For the preparation of propanil β-CD, 0.0384 g of propanil was added while stirring to a solution of β-CD (0.1 g) in water (25 mL) previously warmed to 60 °C. Stirring was maintained for four hours at 60 °C.

### 3.4. X-ray Powder Diffraction (XRD)

A Bruker D8 Advance diffractometer (Karlsruhe, Germany) with a tube anode Cu with its characteristic wavelength of 1.54 Å and a graphite monochromator was used to collect the X-ray diffraction powder patterns. Spectra were recorded at room temperature and 40 kV, and 30 mA. The diffractograms were measured in the 2θ angle range 6–40° and process parameters with the scanning speed 0.04 θ/s.

### 3.5. Fourier Transform Infrared (FT-IR) Spectroscopy

FT-IR analysis was performed on Perkin Elmer Spectrum GX spectrometer (Waltham, MA, USA) apparatus applying a Fourier Transformation of 8 scans between 370–4000 cm^−1^ with a resolution of 1 cm^−1^. Samples were prepared with KBr disks by compressing the powder composed of 1 mg of complex and 100 mg of KBr.

### 3.6. Molecular Docking Studies

The molecular docking studies of the inclusion complexes of β-CD with the three different guests were performed using the package Hex [53], version 3.1.0 for the software Samson, version 2022 R2. The PDB files of β-CD (receptor) and monuron, propanil, and chlorpropham (ligands) were uploaded as inputs into Hex. The parameters used are reported in [54]. All input files were validated using the software Samson. Computations were performed using the shape complementary scoring function, with 16 and 30 expansion orders for the initial and final steps. During each molecular docking calculation, the CD was kept as a fixed truncated cone, and the compounds were allowed to move freely. Hex also performed the structure refinement and the energy minimization of the complexes. Molecular docking results were clustered into different groups based on the root-mean-square deviation values, and the lowest energy host–guest inclusion complex conformation was selected and analyzed. The complexes were analyzed and visualized using the software Samson.

## 4. Conclusions

Pesticide remediation processes from soil and water using CDs as an encapsulating agent depend upon the features of the considered pesticide. The physicochemical properties of the guest molecule change upon the establishment of a host–guest inclusion complex that leads to improvements in terms of solubility and bioavailability. Inclusion often can result in better efficiency or controlled release properties that allow lowering the amount of pesticide spread and, with it, overall environmental remediation.

In addition, the formation of inclusion complexes, including the simple formation of external or non-inclusion complexes (i.e., the physical mixture of β-CD and pesticide), has shown interesting agrochemical properties [55].

For these reasons, we have investigated the interaction between β-CDs and chlorpropham, monuron and propanil, three commonly used pesticides. The characterization has been performed both in solution and in the solid state using different and complementary techniques. The formation of the inclusion complexes in the solid state was confirmed in the three cases by X-ray powder diffractometry and Fourier transform-infrared spectroscopy. The stoichiometry of the prepared complexes was established in solution using the Job plot method to be 1:1, and their binding constants were evaluated by means of UV-Vis spectroscopy. The energy-minimized structures in silico have shown how the toroidal shape of the β-CD nicely accommodates the three pesticides by forming interaction with their aromatic portions. Our data, overall, expand and complement the available literature and are in agreement with previous studies conducted with different techniques [25,51,56] in indicating the β-CD, and in general CDs, as a powerful tool for agrichemical applications.

The distinctive structure of a truncated cone bearing a hydrophobic cavity and hydrophilic surface, along with the ease of their chemistry, render CDs an efficacious instrument in numerous fields with pharmaceuticals [57,58], cosmetics, personal care and toiletry [59], food manufacturing [60] and many others application.

Their ability to include, either partially or entirely, different types of guest molecules, low-cost and easy availability indicate CDs as a possibility to explore for modulating the properties that determine the environmental impact of pesticides.

## Figures and Tables

**Table 1:** The binding constant of Pesticide-β Cyclodextrin complexes evaluated by absorbance measurements and relative R^2^.

Chlorpropham	Monuron	Propanil
K_b_ = 369.9 M^−1^	K_b_ = 292.3 M^−1^	K_b_ = 298.3 M^−1^
R^2^ = 0.9737	R^2^ = 0.9841	R^2^ = 0.9580

**Table 2:** FT-IR bands for the studied molecules and their complexes.

	Chlorpropham	Chlorpropham: β-CD	Monuron	Monuron: β-CD	Propanil	Propanil: β-CD
C=O	1702.01 nm	1735.75 nm			1670.29 nm	1667.98 nm
C=C aromatic	1594.77 nm 1542.4 nm	1596.07 nm 1523.42 nm	1589.06 nm 1514.81 nm	1587.13 nm 1516.74 nm	1590.54 nm 1534.95 nm	1593.23 nm 1535.62 nm
C–Cl	773.68 nm	761.11 nm			814.84 nm	816.05 nm
Metil group			1397.17 nm	1403.92 nm	1474.98 nm	1475.27 nm

## Data Availability

Data used and/or analysed in this study are available from the corresponding author on request.

## Sample Availability

Samples of the compounds used in this study are available from the authors.
